# The impact of preloaded intraocular lens implantation system (TECNIS iTec®) in routine cataract surgery in China: a time-motion analysis

**DOI:** 10.1186/s12886-023-02858-9

**Published:** 2023-03-28

**Authors:** Xudong Song, Jian Zhou, Guangbin Zhang, Songbai Jia, Jun Yuan, Ke Hu, Xinhua Liu, Mingbing Zeng, Zhenyu Wang, Baoying Tan, Xingwei Lu, Ailing Lin, Xiaohan Hu, Jianwei Xuan

**Affiliations:** 1 Beijing Ophthalmology and Visual Science Key Lab, Beijing Tongren Eye Center, Beijing Tongren Hospital, Capital Medical University, Beijing, China; 2 Xijing Hospital, Xian, China; 3 Xiamen Eye Center of Xiamen University, Xiamen, China; 4 The Second Xiangya Hospital of Central South University, Changsha, China; 5 Zhengzhou Second Hospital, Zhengzhou, China; 6 The First Affiliated Hospital of Chongqing Medical University, Chongqing, China; 7 Shenzhen Eye Hospital, Shenzhen, China; 8 Hainan Branch of Zhongshan Ophthalmic Hospital, Hainan, China; 9 Health Economic Research Institute, School of Pharmacy, Sun Yat-sen University, Guangzhou, China

**Keywords:** Preloaded intraocular lens implantation system, Time-motion analysis, Surgical throughput, Hospital revenue, Cataract

## Abstract

**Objective:**

To evaluate the impact on surgical efficiency and labor time cost of preloaded intraocular lens (IOL) implantation system compared with manual IOL implantation system in age-related cataract surgery in China.

**Methods:**

This study was an observational, multicenter, prospective time-motion analysis. IOL preparation time, operation time, cleaning time, number and cost of cataract surgeries in eight participating hospitals were collected. The linear mixed model was used to explore factors associated with the difference in operation time between the preloaded IOL implantation system and the manual IOL implantation system. A time-motion model was constructed to convert the operation time cost saved by using preloaded IOL into economic benefits from hospital and social perspective, respectively.

**Results:**

There were 2,591 cases included in the study (preloaded IOL: 1,591 cases; manual IOL: 1,000 cases). The preloaded IOL implantation system was significant time-saving in both preparation time and operation time compared to the manual IOL implantation system (25.48s vs. 47.04s, *P* < 0.001 and 353.84s vs. 367.46s, *P* = 0.004, respectively). An average total of 35.18s can be saved by using preloaded IOL per procedure. The results of linear mixed model showed that the type of IOL was the main factor leading to the difference in preparation time between preloaded IOL and manual IOL implantation system. By switching from manual IOL to preloaded IOL, the model projected additional 392 surgeries can be performed each year and an increase in revenue of $565,282 per hospital, a 9% increase from hospital perspective. And the annual productivity loss saved by using preloaded IOL was $3,006 in eight hospitals from perspective of society.

**Conclusion:**

Compared with manual IOL implantation system, the preloaded IOL implantation system reduces lens preparation time and operation time, which increases potential surgical volume and revenue, and reduces the loss of work productivity. This study provides real-world evidence to support the advantages of the preloaded IOL implantation system in improving efficiency of ophthalmic surgery in China.

## Introduction

Age-related cataract is an age-related lens opacity disease, which ranks first in the world as a major cause of blindness. With the increase of age, the number of patients with age-related cataract continues to grow as currently observed in China [1, 2]. Findings from a recent meta-analysis showed that the prevalence of age-related cataracts in Chinese males increased from 3.23% at the age of 45 ~ 49 to 65.78% at the age of 85 ~ 89, while the prevalence in females rose from 4.72 to 74.03% in the corresponding age categories [3]. If left untreated, age-related cataract would eventually progress into severe visual impairment or blindness. At present, surgery is the only effective modality to treat cataract, and more than 75% of patients can improve their visual acuity to more than 0.3 after surgery [4, 5].

Cataract surgery refers to the process of removing the opacified lens and then implanting a new intraocular lens (IOL). Successful cataract surgery is reflected in the safety, efficiency, long-term stability of the intraocular lens after implantation, and significant improvement in visual acuity. To achieve these goals, high-quality surgical equipment and IOL are needed. Traditional manual IOL need to be installed under a microscope or naked eyes using tools, such as folding clips and implants, before implantation. A study showed that when using manual IOL, the mean number of IOL physical touch of surgery equipment in each cataract surgery was 4 to 5 [6]. When physician’s assistant or nurse is not skillful in the preparation, wrong installation may occur. At the same time, the loading process increases the time of operation [7] and the risk of IOL contamination, which may lead to various complications, including anterior segment toxicity syndrome, endophthalmitis, severe intraocular tissue injury and severe vision loss or even blindness [8–10]. Conversely, the preloaded IOL implantation system is a non-contact and disposable IOL preloaded system, which avoids exposure to ambient air or physical touch of surgery equipment, minimizing the risk of infection and inflammation caused by contamination. A prospective, multicenter, parallel, single-blind, randomized controlled study in the United States showed that compared with manual IOL implantation system, patients with preloaded IOL implantation system had a smaller corneal incision size and a lower incidence of astigmatism caused by surgery [11].

Time-motion analysis, which is pioneered and developed by American industrial engineers [12], is one of the methods to study scientific management and benefits, and it is a research tool to improve and upgrade the working system. It was used to analyze each action involved in the study and its time in industrial processes in order to find out inefficient links and improve them. Time-motion analysis has also been employed by medical and health organizations to improve their operational efficiency and optimize the use of medical resources. A prospective observational study conducted in the United States, France, and Canada showed that compared to manual IOL implantation system, the preloaded IOL implantation system reduced total procedure time, total surgeon lens time and surgeon delays. The reduced operation time increased the surgical volume and reduced the possibility of IOL contamination and postoperative complications [6]. In China, there is no similar data on the time and motion of preloaded IOL implantation system for age-related contact surgery and the implications of adoption of preloaded IOL implantation system remain to be demonstrated.

This study is based on the time-motion analysis method to evaluate the effects of preloaded IOL implantation system (*Product name: The TECNIS 1-Piece IOL with the TECNIS iTec*^*®*^*Preloaded Delivery System; Manufacturer: Johnson & Johnson Surgical Vision, Inc. China*) and manual IOL implantation system on surgical efficiency and labor time cost for age-related cataract surgery in China. The study also quantified economic benefits associated with the time saved by using preloaded IOL system in terms of potential increased revenue and labor cost savings of the hospitals.

## Methods

### Study design

This study was an observational, multicenter, prospective time-motion analysis. It compared the time spent of using preloaded IOL implantation system (TECNIS iTec^®^) with that of manual IOL implantation system in age-related cataract surgery. The numbers of operations and the cost of cataract surgery were collected through expert surveys. This study was conducted in 8 participating hospitals in 8 provinces in China from May 15, 2020 to June 30, 2021, including Beijing Tongren Hospital (Hospital A), Xijing Hospital (Hospital B), Xiamen Eye Center (Hospital C), The Second Xiangya Hospital of Central South University (Hospital D), Zhengzhou Second Hospital (Hospital E), the First Affiliated Hospital of Chongqing Medical University (Hospital F), Shenzhen Eye Hospital (Hospital G) and Hainan Branch of Zhongshan Ophthalmic Hospital (Hospital H). The study protocol was approved by the hospital ethics committee of the principal investigator (Beijing Tongren Hospital) and informed consent was obtained from all patients.

### Study population

The inclusion criteria were as follows: (1) patients undergoing phacoemulsification with IOL implantation for age-related cataract and (2) the preloaded IOL product used for the operation was The TECNIS1-Piece IOL with the TECNIS iTec^®^ Preloaded Delivery System.

The exclusion criteria were as follows: (1) patients with glaucoma, diabetic fundus disease or other eye diseases or the manual intraocular lens used in surgery which was a disposable intraocular lens implantation system. (2) Abnormal time were screened based on InterQuartile Range [13] (IQR) principle. Abnormal time greater than the sum of the upper quartile and 1.5 times IQR were excluded.

### Data collection and linear mixed model

This study collected the IOL preparation time, operation time and cleaning time of each procedure. The preparation time of the IOL referred to the time when the IOL package was torn open until the IOL was successfully loaded into the implanter. The operation time referred to the time between surgeon’s first cut and completion of the watertight incision. The cleaning time referred to the time required for disinfection of the surgical instruments of a single operation after operation. The total procedural time included IOL preparation time and operation time. In addition, the operation-related information was collected, such as credentials of IOL preparation and cleaning staff (surgeon, surgical assistant, nurse, professional cleaning specialist) and Emery nuclear hardness grading (grade I, II, III, IV, V). Observers recorded the time spent in each step of the operation using a timer. Before conducting the study, the observers were trained on time measurement and use of measurement tools to ensure the reliability of data.

Through a survey of 8 experts from 8 participating hospitals, data on number of age-related cataract surgery performed each year and the procedure cost were collected.

Based on the potential confounding factors suggested by clinical experts, the linear mixed model (LMM) was employed to test whether there was a statistically significant difference in time between the preloaded IOL and the manual IOL implantation systems. In the model, each center was considered a random effect variable and other factors were considered fixed effect variables to identify factors that were significantly associated with the time difference between the preloaded IOL and the manual IOL implantation systems. Fixed effect parameters for preparation time included preparers and type of IOL, and for operation time included nuclear grade and type of IOL.

$$\begin{array}{c}\log (time) = Y = {\beta _0} + {x_1}{\beta _{type\,\,of\,\,IOL}} + {x_2}{\beta _{preparation\,\,staff}}\\+ {x_3}{\beta _{nuclear\,\,grade\,}} + {u_4}{\gamma _{hospital}} + \in \end{array}$$

where β is fixed effect parameter, γ is random effect parameter.

### Time-motion model

**Fig. 1:**
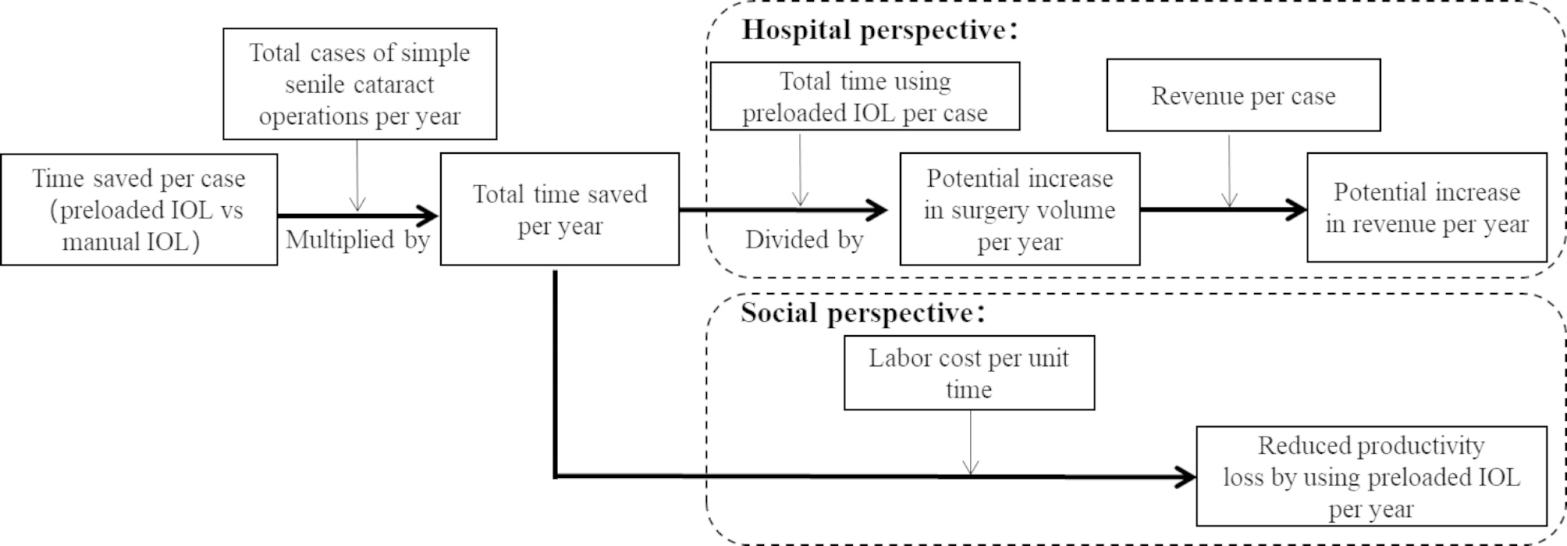
Time-motion model structure

A time-motion model (Fig. 1) was constructed to convert the operation time saved by using preloaded IOL into economic benefits. The Chinese currency was converted to US dollars (exchange rate 1 USD = 6.5249 CNY; 31 December 2020). From perspective of hospital, the potential increase in surgical volume attributed to preloaded IOL in the participating hospital was evaluated. Under the assumption that operation efficiency remains unchanged, if the total time saved per operation day exceeds the operation time by using the preloaded IOL, one or more operations is projected to be performed on that day.

From perspective of society, the increase in work productivity of medical staff by using preloaded IOL was evaluated under the condition of constant surgical volume.

### Statistical analysis

Descriptive results were presented as mean and standard deviation for continuous variables. For categorical variables, frequencies and percentages were calculated. Statistical comparisons were performed using Student’s t-test or Wilcoxon rank-sum test for continuous variables as appropriate and Pearson χ2 test or Fisher exact test as appropriate for categorical variables. All tests were 2-sided and performed at a 5% α-level. Statistical analysis was performed using the R software (Version 3.5.3).

## Results

**Fig. 2:**
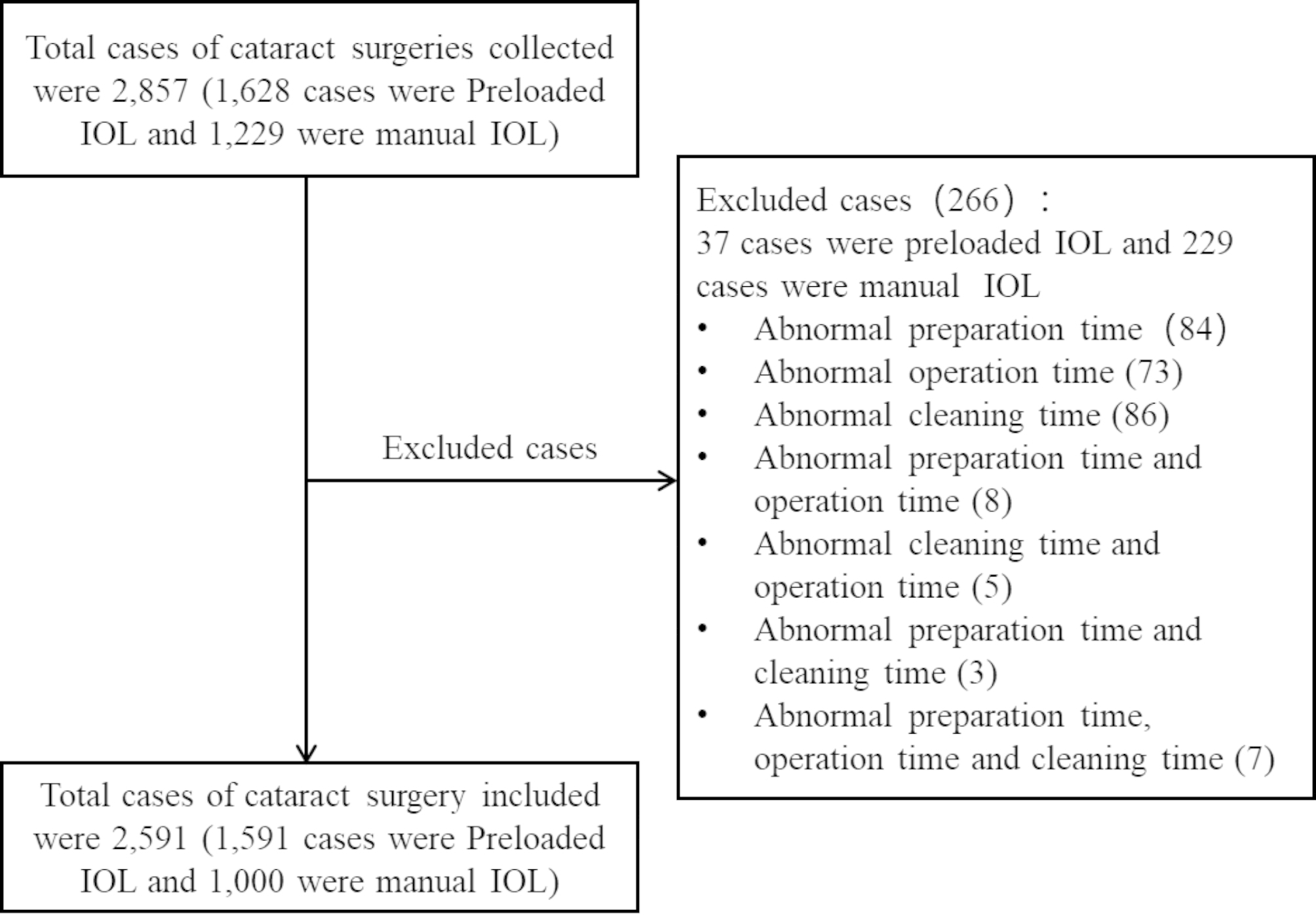
Inclusion and exclusion flow chart of age-related cataract surgery

In this study, a total of 2,857 cases using preloaded IOL implantation system and manual IOL implantation system were collected from eight participating centers. Upon excluding 266 cases which were deemed to be outliers per pre-determined criteria, 2,591 cases were included in the final analysis, including 1,591 cases using preloaded IOL implantation system and 1,000 cases using manual IOL implantation system (Fig. 2).

### Preparation time and operation time

**Fig. 3:**
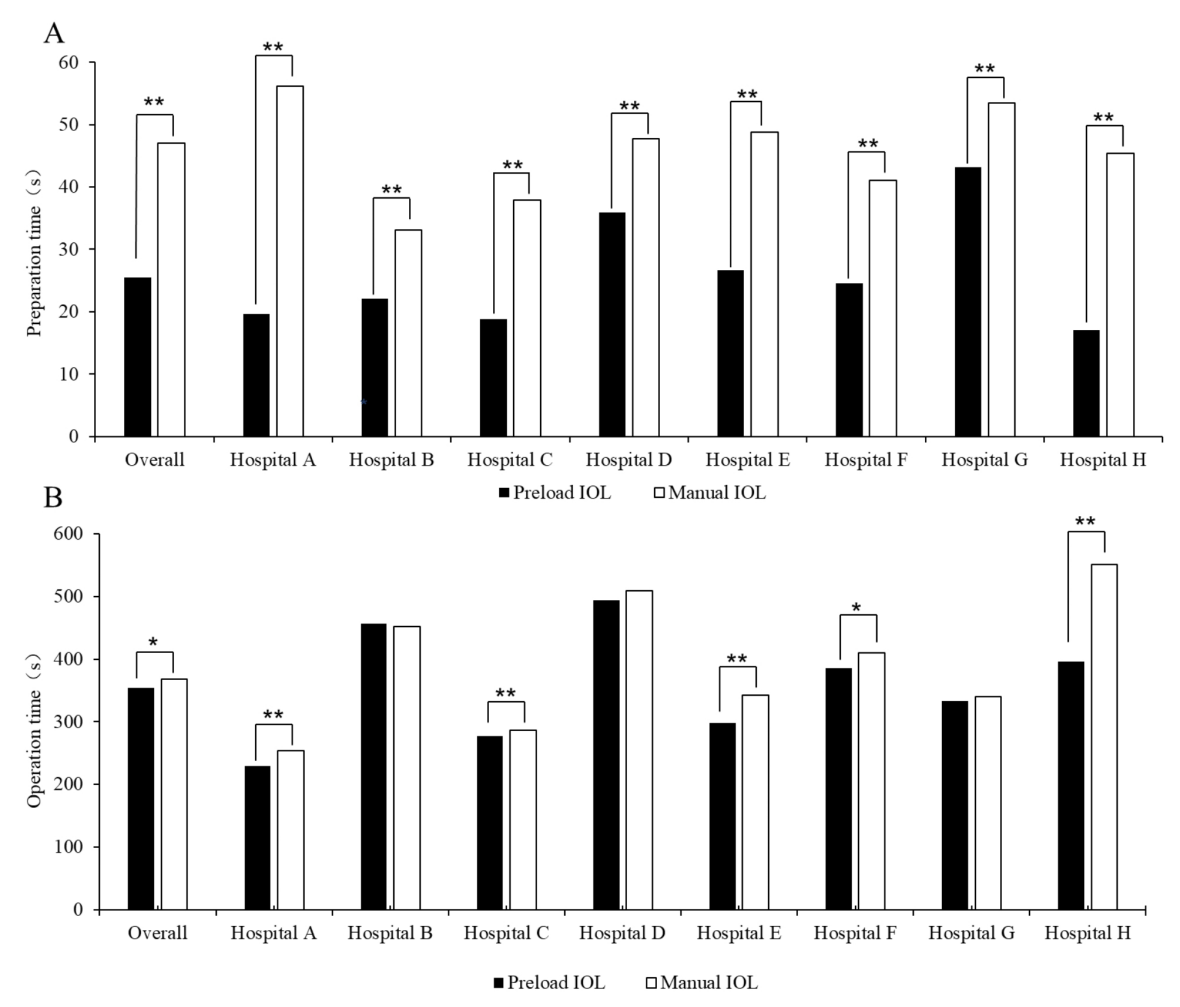
Preparation time and operation time by hospitals **A** Preload and manual IOL preparation time by hospitals. **B** Preload and manual IOL operation time by hospital. *P < 0.05; **P < 0.001

The mean total procedural time of each procedure using preloaded IOL implantation system and manual IOL implantation system were 379.32 and 414.50 s, respectively. Compared with manual IOL implantation system, an average of 35.18s were saved by using preloaded IOL implantation system, with 8.54% reduction (Fig. 3). Owing to high heterogeneity in the processes and methods of cleaning IOL by different hospitals, the cleaning time was not included in the total time as a whole but analyzed separately as a subgroup.

There was a significant difference in the mean preparation time between the preloaded IOL implantation system and the manual IOL implantation system (25.48s vs. 47.04s, P < 0.001), with 45.83% reduction in IOL preparation time using preloaded IOL implantation system. The preloaded IOL implantation system was significant time-saving in the part of preparation time compared to the manual IOL implantation system. Across participating hospitals, the preparation time shortened by using preload IOL system varied from 19.20 to 65.09% (Table 1).

**Table 1:** Preload and manual IOL preparation time by hospitals

Hospital	Preload (s)		Manual (s)		Difference^a^	*P*-value
	**N**	**Mean** ± **SD**	**N**	**Mean** ± **SD**		
Overall	1591	25.48 ± 11.03	1000	47.04 ± 14.72	-45.83%	< 0.001**
Hospital A	253	19.59 ± 5.49	308	56.11 ± 12.26	-65.09%	< 0.001**
Hospital B	295	22.12 ± 8.51	98	33.15 ± 11.73	-33.27%	< 0.001**
Hospital C	186	18.86 ± 2.50	119	37.92 ± 2.31	-50.26%	< 0.001**
Hospital D	151	35.95 ± 4.65	149	47.70 ± 6.17	-24.63%	< 0.001**
Hospital E	230	26.64 ± 10.51	60	48.81 ± 15.22	-45.42%	< 0.001**
Hospital F	141	24.60 ± 8.37	138	41.03 ± 17.69	-40.04%	< 0.001**
Hospital G	184	43.19 ± 8.19	60	53.45 ± 8.31	-19.20%	< 0.001**
Hospital H	151	17.08 ± 6.91	68	45.44 ± 20.47	-62.41%	< 0.001**

^**a**^**Difference**: **Preload IOL vs. Manual IOL no bold**

*P < 0.05; **P < 0.001

Overall, there was also a statistically significant difference in the mean operation time between the preloaded IOL implantation system and the manual IOL implantation system (353.84s vs. 367.46s, *P* = 0.004). Using the preloaded IOL implantation system shortened the operation time by 3.71%. Across participating hospitals, Hospital B/Xijing Hospital showed no significant differences in the mean operation time comparing preload IOL with manual IOL, while other hospitals had shorter operation time with preloaded IOL implantation system varying from 2.10 to 28.11% reduction (Table 2).

**Table 2:** Preload and manual IOL operation time by hospital

Hospital	Preload (s)		Manual (s)		Difference^a^	*P*-value
	**N**	**Mean** ± **SD**	**N**	**Mean** ± **SD**		
Overall	1591	353.84 ± 110.28	1000	367.46 ± 126.82	-3.71%	0.004*
Hospital A	253	229.17 ± 55.64	308	253.86 ± 52.30	-9.73%	< 0.001**
Hospital B	295	456.65 ± 89.81	98	452.37 ± 97.88	0.95%	0.690
Hospital C	186	277.15 ± 22.00	119	286.02 ± 10.17	-3.10%	< 0.001**
Hospital D	151	493.79 ± 75.10	149	508.76 ± 76.07	-2.94%	0.087
Hospital E	230	298.57 ± 68.59	60	342.91 ± 73.51	-12.93%	< 0.001**
Hospital F	141	385.67 ± 55.50	138	410.33 ± 74.16	-6.01%	0.002*
Hospital G	184	332.82 ± 26.87	60	339.97 ± 55.74	-2.10%	0.184
Hospital H	151	396.46 ± 93.31	68	551.49 ± 127.63	-28.11%	< 0.001**

^**fora**^**Difference**: **Preload IOL vs. Manual IOL no bold**

*P < 0.05; **P < 0.001

In this study, the cleaning time was analyzed by subgroup analysis and only the Hospital C and F were included because of their standardized cleaning protocols for both the preloaded IOL and manual IOL systems. Compared with the manual IOL system, the cleaning time of preloaded IOL system was significantly shorter in both hospitals (*P* < 0.001); For hospital C, the cleaning time of preloaded IOL and manual IOL was 113.27 and 142.34 s and for Hospital F, the cleaning time was 20.25 and 26.88 s for the respective IOL system.

### Linear mixed models for IOL preparation time and operation time

The results of linear mixed model showed that in the overall analysis, the type of IOL was the significant factor leading to the difference in preparation time between preloaded IOL implantation system and manual IOL implantation system. And nuclear hardness grading and IOL type were the significant factors leading to the difference in operation time between preloaded IOL implantation system and manual IOL implantation system. Compared with manual IOL implantation system, preloaded IOL implantation system saved more preparation time and operation time, and the difference was statistically significant (Tables 3 and 4).

**Table 3:** Linear Mixed Model for IOL preparation time

Parameter	Coefficient	Std. Error	*P*-value
Preparer: Nurse	0.00	NA	NA
Preparer: Surgical assistant	0.18	0.16	0.270
Preparer: Surgeon	0.25	0.16	0.129
Manual IOL System	0.00	NA	NA
Preloaded IOL System	-0.64	0.01	<0.001*

* Represents statistically significant

**Table 4:** Linear Mixed Model for operation time

Parameter	Coefficient	Std. Error	*P*-value
Grade II nuclear	0.00	NA	NA
Grade I nuclear	0.03	0.04	0.383
Grade III nuclear	0.05	0.01	<0.001*
Grade IV nuclear	0.21	0.01	<0.001*
Grade V nuclear	0.32	0.03	<0.001*
Manual IOL System	0.00	NA	NA
Preloaded IOL System	-0.07	0.01	<0.001*

* Represents statistically significant

### Annual potential increase in surgical volume and revenue

The mean annual cataract surgical volume per hospital was 6,883, of which age-related cataract surgery accounted for 61%. The average revenue of each age-related cataract surgery was $1,442.

From hospital perspective, assuming that all age-related cataract surgeries in each hospital are to be carried out with preloaded IOL onward, additional 392 procedures can be performed and an increase in annual revenue of $565,282 can be generated, i.e., an increase of 9% (Table 5).

The average annual salary of medical staff in public hospitals was $16,690 in 2020 [14], and the number of working days in 2020 was 251 days. From perspective of society, it is assumed that all age-related cataract operations in eight hospitals used manual IOL, and the productivity loss attributed to the use of manual IOL was $0.081 per procedure. The aggregated annual productivity loss associated with use of manual IOL was $3,006 in eight hospitals.

**Table 5:** Annual potential increase in surgery volume and revenue

Economic benefits from hospital perspective	Value
Potential increase in operations	
Age-related cataract surgery volume with all using manual IOL: per year (cases)	4,231
Age-related cataract surgery volume with all using preloaded IOL: per year (cases)	4,623
Preloaded IOL vs. manual IOL: potential annual increase of surgery volume (cases)	392
Preloaded IOL vs. manual IOL: potential annual increase rate (%)	9%
**Potential increase in revenue**	
Senile cataract surgery revenue with all use manual IOL: per year ($)	6,101,542
Senile cataract surgery revenue with all use preloaded IOL: per year ($)	6,666,824
Preloaded IOL vs. manual IOL: potential annual increase of revenue ($)	565,282
Preloaded IOL vs. manual IOL: potential annual increase rate (%)	9%

## Discussion

Cataract is one of the major causes of blindness. With increasing age of global population, the number of patients with visual impairment caused by cataract may have reached 50 million by 2020 [15, 16]. Surgery remains the effective intervention to restore vision. In recent years, the improved materials, design and function of IOLs further drive the demand of cataract replacing surgeries [17–19].

The preloaded IOL has been demonstrated for its safety and effectiveness, long-term stability and excellent visual quality [6, 11, 20]. Surgeons and surgical technicians are more satisfied with the use of preloaded IOL, especially for the ease of lens preparation, the number of steps required and the total implantation time [20–23]. Additionally, the preloaded IOL offers several benefits over reusable, manual IOL, including a reduced chance of IOL damage during loading, shortened surgery time, the need for fewer surgical instruments, lower risk for contamination, and elimination of other manual setting errors, reduced incision size and quicker recovery [11, 20, 24]. At the same time, the particularities of preloaded IOL reflected in two aspects. On the one hand, it takes time for surgeons to learn and master how to use this preloaded IOL. On the other hand, the pushers of preloaded IOL are disposable and non-sterilizable, which is not in line with the sustainability concept.

As the population ages at an accelerated rate in China and worldwide, the number of cataract patients is projected to double within the next 30 years, presenting significant challenges for healthcare systems and limited resources. For a typical cataract surgery, the typical operation duration is as brief as 10–20 min. As a result, even minor improvements in surgical procedures and operating methods can quickly lead to substantial improvements in the number of cataract surgeries performed and the overall efficiency of the healthcare system. The personnel and operating room are both valuable and costly. Maximizing the efficiency of staff and operating rooms could allow for more patients to receive medical care. In this research, the effects on efficiency and revenue of adopting a preloaded IOL (TECNIS iTec^®^ Preloaded Delivery System) were analyzed to determine the impact on the number of cataract surgeries performed and hospital revenue of transitioning from manual IOL to preloaded IOL. This study showed that application of the preloaded IOL significantly reduced the preparation time and operation time. The linear mixed model analysis indicated that the key factor affecting preparation time and operation time was the type of IOL. And it was estimated that switching from manual IOL to preloaded IOL could increase the surgery volume and revenue without additional staffs.

To our knowledge, this is the first study to evaluate the time savings and economic benefits from a hospital revenue perspective and a social productivity perspective of preloaded IOL in China with a large sample size. Our results were consistent with previous findings. A single-center, prospective and observational study in northwest China showed that the surgeon lens time was 0.7 min using preloaded IOL, which was a 46.2% reduction compared with manual IOL (1.3 min) [25]. By switching from manual IOL to preloaded IOL, annual cataract surgery volume would increase by 5.2%, accompanied by an increase in revenue by $284,352 [25]. A study conducted in the United States, France and Canada found that the use of preloaded IOL can reduce mean total procedure time by 6.2-12% [6]. The annual surgical volume would increase by 9.9% when switching from manual IOL to preloaded IOL [6]. Another study conducted in France and Spain showed that the implantation time of preloaded IOL was similar to that of manual IOL (12.9s vs. 12.2s), but the preparation time of preloaded IOL was shorter than that of manual IOL with a 49.3% reduction (30.3s vs. 59.8s) [21]. A study conducting in New Zealand and Australia found that the mean estimated time-savings per procedure was 54.9s compared with non-preloaded delivery systems [20]. The preloaded IOL has the design advantages for elimination of the need for loading IOL into the introducer and combining the introducer with the pusher, which reduced number of preparation steps and improves intraoperative efficiency. In contrast to previous studies, our study also showed that the preloaded IOL significantly saved more preparation time and operation time compared to manual IOL upon controlling for effects of preparer type, surgery staff type, and patient nuclear hardness grade on time. Other potential influential factors, such as surgeons with varying levels of ophthalmic surgical experience, adhesion between haptics and optics will be collected in the future study. In addition, our study also measured the labor loss of surgeons and surgical technicians from the perspective of society.

In the current study, the calculated potential increase in revenue was derived by three factors: the time saved by preloaded IOL system, the cost per age-related cataract surgery, and volume of age-related cataract surgery. As the preparation process and surgical procedure may vary by sites, we used average time saved in eight participating study sites to estimate the potential increased revenue, which likely is more representative of typical clinical settings in China. Clinical experts survey indicated that there was no difference in surgical costs between preloaded IOL and manual IOL, with no additional instrumentation costs and less pushers used for preloaded IOL surgery. The surgical volume from participating hospitals was also obtained from the clinical experts’ survey, which was based on the real-world data from their respective hospital electronic information system. Taken together, the results of our study appeared to be robust.

Potential limitations should be taken into consideration when interpreting the findings of this study. This study was an observational, non-interventional study, which was not possible to control for unobserved external factors in participating hospitals (e.g., surgical procedure flow, cataract case mix, patient characteristics, etc.). The implication on surgical volume and hospital revenue was projected based on historical record not a direct observation. In fact, the annual surgical volume of 8 hospitals from July 2020 to July 2021 was significantly lower compared to the years prior due to the impact of the COVID epidemic. As a result, the potential increase in revenue and loss of labor costs may have been underestimated. Finally, this study only focused on the economic impact of the application of preloaded IOL system without follow-up on clinical and patient-centered outcomes, such as patient quality of life and satisfaction with preloaded or manual IOL before and after the implementation of cataract patients. However, these limitations did not affect the conclusions of this study which focused on the surgical efficiency comparing the preloaded IOL system with manual IOL system.

## Conclusion

Compared with manual IOL implantation system, the preloaded IOL implantation system reduces lens preparation time and operation time, which is projected to significantly increase potential surgical volume and revenue, and reduces the loss of work productivity. This study provides real-world evidence to support the advantages of the preloaded IOL implantation system in improving the efficiency of ophthalmic surgery in China.

## Data Availability

All related data were displayed in the manuscript. Further information regarding the data can be obtained by contacting the corresponding authors.

## Abbreviations

IOL: intraocular lens
LMM: linear mixed model
IQR: InterQuartile Range
